# Causes, patterns and severity of androgen excess in 487 consecutively recruited pre- and post-pubertal children

**DOI:** 10.1530/EJE-18-0854

**Published:** 2018-12-19

**Authors:** Jan Idkowiak, Yasir S Elhassan, Pascoe Mannion, Karen Smith, Rachel Webster, Vrinda Saraff, Timothy G Barrett, Nicholas J Shaw, Nils Krone, Renuka P Dias, Melanie Kershaw, Jeremy M Kirk, Wolfgang Högler, Ruth E Krone, Michael W O’Reilly, Wiebke Arlt

**Affiliations:** 1 Institute of Metabolism and Systems Research, University of Birmingham; 2 Department of Endocrinology and Diabetes, Birmingham Women’s and Children’s Hospital NHS Foundation Trust; 3 Centre for Endocrinology, Diabetes and Metabolism, Birmingham Health Partners; 4 Department of Clinical Biochemistry, University Hospitals Birmingham NHS Foundation Trust, Birmingham, UK; 5 Academic Unit of Child Health, Department of Oncology & Metabolism, University of Sheffield, Sheffield, UK; 6 Department of Pediatrics and Adolescent Medicine, Johannes Kepler University Linz, Linz, Austria

## Abstract

**Objective:**

Androgen excess in childhood is a common presentation and may signify sinister underlying pathology. Data describing its patterns and severity are scarce, limiting the information available for clinical decision processes. Here, we examined the differential diagnostic value of serum DHEAS, androstenedione (A4) and testosterone in childhood androgen excess.

**Design:**

Retrospective review of all children undergoing serum androgen measurement at a single center over 5 years.

**Methods:**

Serum A4 and testosterone were measured by tandem mass spectrometry and DHEAS by immunoassay. Patients with at least one increased androgen underwent phenotyping by clinical notes review.

**Results:**

In 487 children with simultaneous DHEAS, A4 and testosterone measurements, we identified 199 with androgen excess (140 pre- and 59 post-pubertal). Premature adrenarche (PA) was the most common pre-pubertal diagnosis (61%), characterized by DHEAS excess in 85%, while A4 and testosterone were only increased in 26 and 9% respectively. PCOS was diagnosed in 40% of post-pubertal subjects, presenting equally frequent with isolated excess of DHEAS (29%) or testosterone (25%) or increases in both A4 and testosterone (25%). CAH patients (6%) predominantly had A4 excess (86%); testosterone and DHEAS were increased in 50 and 33% respectively. Concentrations increased above the two-fold upper limit of normal were mostly observed in PA for serum DHEAS (>20-fold in the single case of adrenocortical carcinoma) and in CAH for serum androstenedione.

**Conclusions:**

Patterns and severity of childhood androgen excess provide pointers to the underlying diagnosis and can be used to guide further investigations.

## Introduction

Androgen excess in childhood may present with a variety of symptoms and is thought to have a broad spectrum of underlying pathologies (1). Premature pubic and axillary hair growth, change in body odor and transient growth acceleration are typical presenting signs in pre-pubertal children (2); menstrual disturbances with hirsutism are presenting features in post-pubertal girls (3). In the vast majority of affected pre-pubertal children, PA is the underlying diagnosis, whereas adolescent polycystic ovary syndrome (PCOS) is the leading cause of androgen excess in pubertal girls after menarche (1, 2, 4). Importantly, the diagnosis of PA and PCOS require exclusion of other causes of androgen excess such as inborn steroidogenic enzyme defects, most commonly congenital adrenal hyperplasia (CAH), precocious puberty or potentially malignant virilizing adrenal tumors, with the latter being extremely rare in childhood (1, 5, 6).

To reach a conclusive diagnosis in a child presenting with androgen excess can be challenging. Detailed history, including onset, acuity, severity and progression of symptoms and a thorough clinical examination, followed by hormonal investigations, bone age assessment, and, where appropriate, imaging studies, are part of the clinical work-up (1, 7). The extent of investigations required usually depends on the severity and acuity of presenting symptoms, and clinicians tend to tailor those investigations depending on the clinical presentation and the severity of biochemical androgen excess (7). However, there is a paucity of data from larger cohorts delineating patterns and severity of childhood androgen excess considered predictive for both common and rare underlying pathologies.

Physiologically low circulating androgen levels in children and the widespread use of radioimmunoassays, which are prone to cross-reactivity and low sensitivity, limit the diagnostic accuracy of the measurement of the serum concentrations of the active androgen testosterone and the androgen precursors androstenedione (A4) and DHEAS, in particular when measured in isolation (8, 9). Liquid chromatography–tandem mass spectrometry (LC–MS/MS) analysis of serum steroids has emerged as a highly sensitive analytical tool, in particular when measuring low-abundance steroids in children (10, 11). To date, there is a dearth of data on LC–MS/MS-based androgen measurements in childhood androgen excess conditions.

We have recently reported the utility of simultaneous measurement of serum DHEAS, A4 and testosterone in determining causes, patterns and severity of androgen excess in a large sample of adult women recruited in a single center (12), generating useful guidance for clinicians to predict non-PCOS pathology in adult women presenting with androgen excess. In this study, we aimed to develop the evidence base for a rational approach to childhood androgen excess. To this end, we analyzed a large cohort of consecutively recruited children from a single tertiary referral center to uncover the signatures of distinct conditions underlying childhood androgen excess.

## Patients and methods

### Subjects and clinical protocol

Institutional review board approval for retrospective data review was obtained from Birmingham Women’s and Children’s Hospital (BWCH) NHS Foundation Trust (reference: CARMS-00935). We included all children who had undergone measurements of serum testosterone, A4 and DHEAS as part of routine clinical care at BWCH between 1st January 2013 and 1st June 2017 (*n* = 1525), identifying 487 who underwent simultaneous measurement of DHEAS, A4 and testosterone. Samples were collected at variable times during the day in the context of outpatient clinic appointments. Patients with at least one serum androgen concentration increased above the Tanner stage-specific reference range (*n* = 199) underwent further clinical phenotyping by case note review, extracting information on clinical presentation, medical history, height, weight, BMI, bone age, ethnicity and the underlying cause of androgen excess, as supported by clinical, biochemical and radiological findings in each case with final review by a board-certified pediatric endocrinologist (V S, T G B, N J S, N K, R P D, M K, J K, W H, R E K). Reference data for standardized BMI (BMI SDS) were obtained from the British 1990 dataset (13).

### Serum androgen measurements

Biochemical androgen excess was defined as a serum concentration increased above a local, age-specific reference range (for DHEAS) or above the Tanner stage-specific normative reference range as reported in 14 for A4 and testosterone. Serum A4 and testosterone were analyzed by liquid chromatography–tandem mass spectrometry on a Shimadzu Prominence XR UPLC coupled to a Sciex 6500 Triple Quad mass spectrometer as described previously (12). Briefly, samples are analyzed by liquid–liquid extraction following addition of deuterated internal standards and separated chromatographically using an isocratic elution profile, ionized using positive atmospheric pressure chemical ionization and detected according to compound-specific transitions. Serum DHEAS was analyzed using the Roche competitive electrochemiluminescence immunoassay on the Roche Cobas c702 analyzer (12).

### Statistical analysis

GraphPad Prism was used for statistical analysis and generation of graphs. Data were expressed as median and first and third quartile, unless otherwise stated. The Mann–Whitney *U* test was used for comparison between two groups (pre- and post-pubertal). Spearman rank correlation was employed to assess correlation between two non-evenly distributed variables. Statistical significance was set at *P* < 0.05.

## Results

### Description of the cohort and diagnostic spectrum

**Figure 1:**
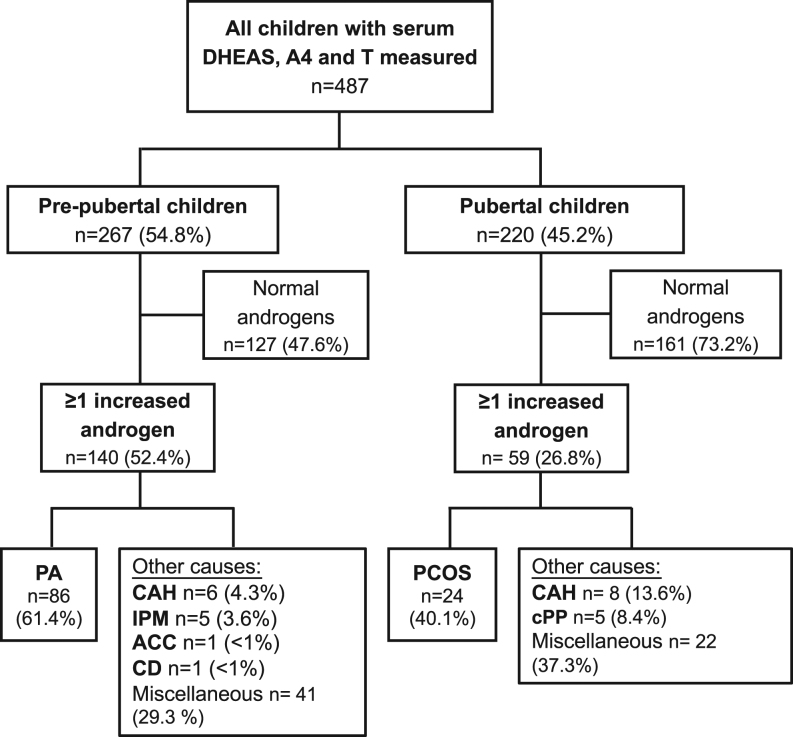
Flowchart of the distribution of diagnoses according to pre-pubertal vs post-pubertal status in 487 children who underwent simultaneous measurement of serum DHEAS, A4 and testosterone. ACC, adrenocortical carcinoma; CAH, congenital adrenal hyperplasia; CD, Cushing’s disease; cPP, central precocious puberty; IPM, isolated premature menarche; PA, premature adrenarche; PCOS, polycystic ovary syndrome.

A total of 1525 children had at least one serum concentration of DHEAS, A4 or testosterone measured during the study period. In 487 children (31.9%), all three androgens had been measured simultaneously and were taken forward as the analysis cohort (Fig. 1). When applying age-defined cut-offs, 255 children (52.4%) had at least one increased serum androgen; however, when applying Tanner stage-defined cut-offs, thus taking into account pubertal development, only 199 children (40.9%) had androgen excess (Fig. 1). Those 199 children were phenotypically further characterized as described in the ‘Methods’ section. There was a pre-dominance of girls (Table 1). The median BMI SDS was increased in the overall cohort and higher in the pubertal than in the pre-pubertal group (Table 1). The overall cohort was ethnically diverse, with the two largest group comprising Caucasian children (44.2%) and children of South Asian ethnicity (38.7%) (Table 1).

**Table 1:** Baseline demographics of 199 children with biochemical androgen excess as defined by serum concentrations above the reference range for at least one of three measured androgens (DHEAS, androstenedione and testosterone).

	Total	Pre-pubertal	Pubertal
Patients with ≥1 of 3 androgens increased	199	140 (70.3%)	59 (29.7%)
Age (years; median (Q1, Q3))	8.3 (6.8, 13.3)	7.4 (4.6, 8.5)	14.9 (13.4, 15.6)
Gender (%)			
Girls	141 (70.8)	89 (63.6)	52 (88.1)
Boys	58 (29.2)	51 (36.4)	7 (11.9)
BMI (kg/m^2^; median (Q1, Q3))	19.5 (16.4, 25.1)	17.9 (15.9, 22.0)	26.8 (19.8, 31.0)
BMI SDS (median (Q1, Q3))	1.35 (0.2, 2.6)	1.26 (−0.1, 2.5)	1.88 (0.6, 2.7)
Ethnicity* (%)			
Caucasian	91 (44.2)	64 (44.2)	27 (44.2)
South Asian	76 (38.7)	50 (36.9)	26 (44.1)
Afro-Caribbean	16 (8.0)	13 (9.4)	3 (4.9)
Mixed background	3 (1.5)	2 (1.5)	1 (1.6)
Other	1 (0.5)	0	1 (1.6)
Unknown	13 (6.5)	11 (7.2)	3 (4.9)

United Kingdom Census 2011, Office for National Statistics (15).

*Ethnicity distribution pattern in the Birmingham area is: Caucasian 58.0%, Asian 26.6% (South Asians 22.5%, other Asians 4.08%), Afro-Caribbean 9.0%, mixed 4.4% and other 2.0%.

The overall majority of pre-pubertal children had a diagnosis of PA (*n* = 86; 61%), while at pubertal age, PCOS was the most common diagnosis (*n* = 24; 40%) (Fig. 1). In both subgroups, rare and very rare underlying causes of androgen excess were also identified, including CAH (7.0%), isolated premature menarche (IPM; 2.5%), central precocious puberty (cPP; 2.5%) and one case each of adrenocortical carcinoma (ACC) and Cushing’s disease (CD). In about one third of cases, miscellaneous diagnoses and features were reported or no associated diagnosis was made (Fig. 1 and Supplementary Table 1, see section on supplementary data given at the end of this article).

The majority of the 86 children with PA were girls (79%). Girls with PA presented at a median age of 7.2 years, boys at 8.2 years. Forty-one PA children (48%) were of Caucasian ethnicity, 31 children (26%) of South-Asian and 12 children (14%) of Afro-Caribbean descent; the general population in the local area has been recorded as 58.0% Caucasian, 22.5% South Asian and 9% Afro-Caribbean (15). There were no notable gender differences with regards to clinical presentation (Supplementary Table 2), although acne was rare in boys. The median bone age advancement in the PA cohort was 1.88 years (IQR: 1.16, 2.33). None of the subjects had notable co-morbidities or were on any regular medication.

In subjects diagnosed with adolescent PCOS, the median age at presentation was 15.2 years (IQR: 13.1, 15.7). The majority was of South-Asian ethnicity (*n* = 18; 75%); three were Caucasian (12.5%) and two girls were African-Caribbean (8%). The diagnosis of PCOS was established on the basis of the presence of an irregular menstrual cycle and biochemical features of androgen excess. In addition, 63% (*n* = 15) girls also had clinical signs of androgen excess (hirsutism and/or acne) and seven subjects complained about weight gain (29%). The median BMI of the PCOS subjects was 27.1 kg/m^2^ (2.24 SDS; IQR: 0.91, 2.58). None of the PCOS subjects had significant co-morbidities, and they were not on any anti-androgenic medication or metformin at presentation.

All 14 CAH patients had a genetically confirmed diagnosis of 21-hydroxylase (CYP21A2) deficiency; 12 had the classic salt-wasting form and two subjects had non-classic CAH. All classic CAH cases were on steroid replacement therapy; the non-classic cases were not on medication.

We identified five girls with IPM and five with central precocious puberty (cPP). All IPM cases presented with isolated vaginal bleeding. All girls with cPP presented with early breast development and additional signs of androgen excess (premature pubarche *n* = 4; early development of axillary hair *n* = 3; acne *n* = 2). In extremely rare cases, one boy presented with ACC and one boy with CD. The ACC case presented at the age of 1.8 years with peripheral precocious puberty. The boy with CD presented at 13 years of age with weight gain, easy bruising, headaches and typical Cushingoid appearance and was found to have an ACTH-producing pituitary micro-adenoma.

### Patterns of androgen excess according to the underlying diagnosis

**Figure 2:**
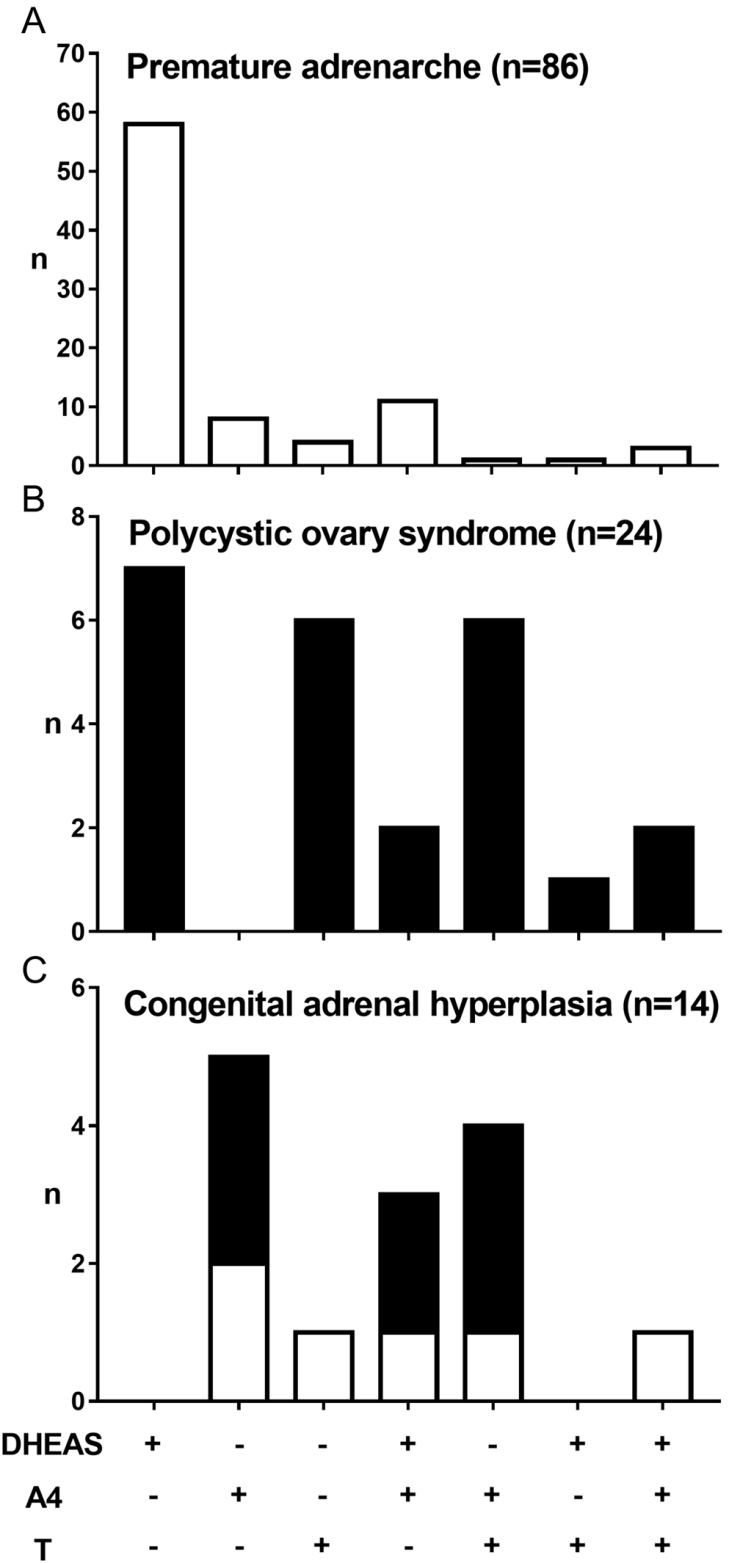
Distribution of serum androgen excess patterns in (A) children with premature adrenarche, (B) girls with polycystic ovary syndrome and (C) children with congenital adrenal hyperplasia. White bars represent pre-pubertal subjects, black bars post-pubertal subjects.

Isolated DHEAS excess was the most common biochemical presentation in children presenting with PA (*n* = 58; 67.4%) (Fig. 2A). Eleven PA subjects (12.8%) had a combination of DHEAS and A4 excess. Isolated A4 or testosterone excess was rare in PA and only found in nine (10.5%) and four (4.7%) subjects. A pattern with all three androgens elevated was only observed in three PA subjects (3.5%). In adolescent PCOS, patients equally frequently presented with isolated DHEAS excess (29%), isolated testosterone excess (25%) or increased serum concentrations of both A4 and testosterone (25%) (Fig. 2B).

Children with CAH predominantly presented with A4 excess (86%); testosterone and DHEAS were increased in 50 and 33% respectively (Fig. 2C). Isolated DHEAS was never observed in CAH children, and only one pre-pubertal CAH patient presented with isolated testosterone excess and increases in all three androgens respectively. In the ACC case, both DHEAS and A4 were increased, and the CD case had isolated A4 excess.

### Severity of androgen excess

**Figure 3:**
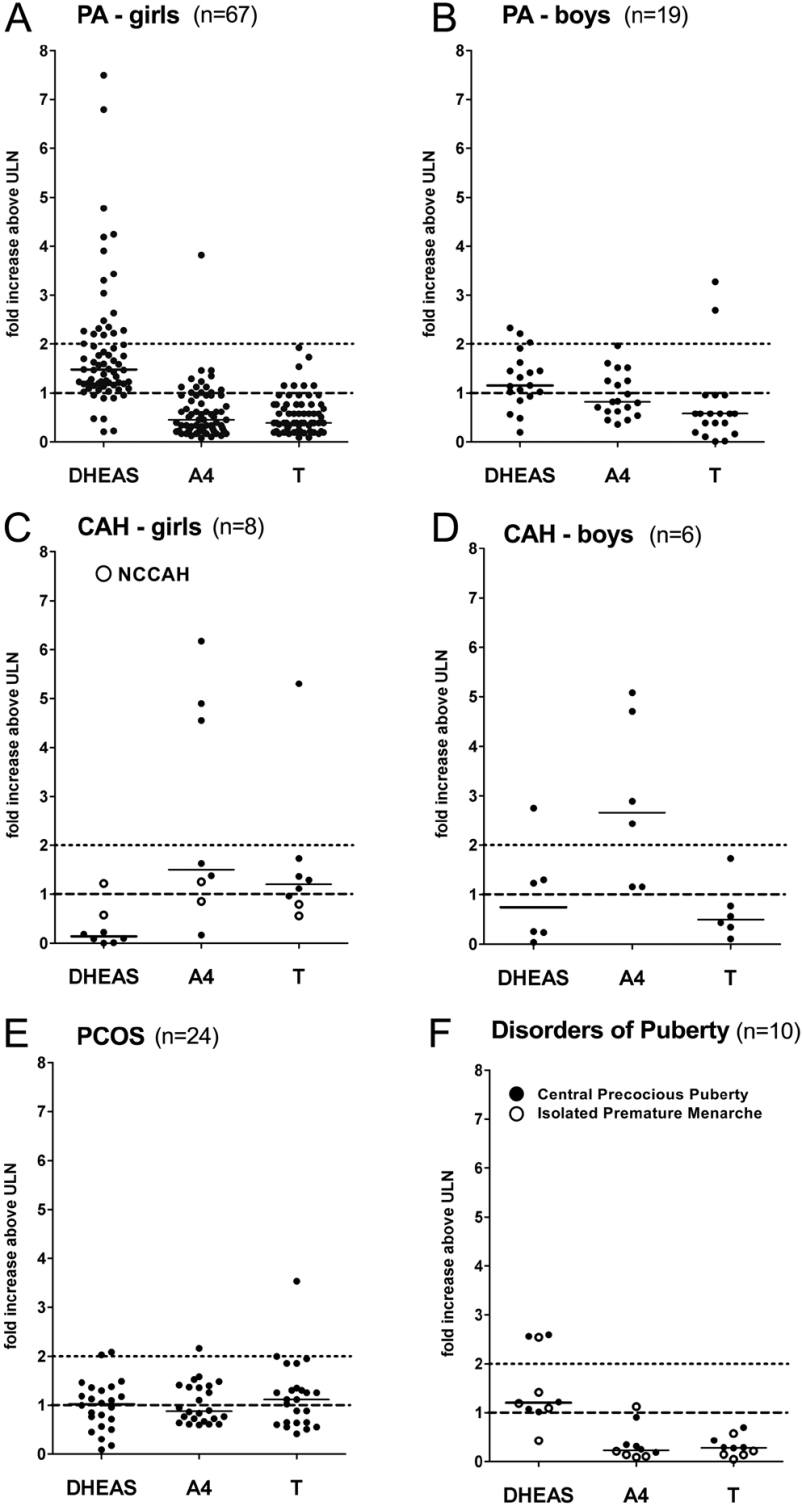
Severity of androgen excess according to diagnosis (A–F) and serum androgen measured. (A) premature adrenarche (PA) in girls; (B) PA in boys; (C) congenital adrenal hyperplasia (CAH) in girls (closed circles: classic CAH; open circles: non-classic CAH); (D) CAH in boys; (E) polycystic ovary syndrome (PCOS); (F) disorders of puberty (closed circles: central precocious puberty; open circles: isolated premature menarche). Androgen excess levels are represented as ‘fold increase above upper limit of normal, ULN’; levels above ‘1’ therefore indicate androgen excess, indicated by the black interrupted line. An arbitrary defined cut-off for severe androgen excess from 2-fold ULN is indicated by a black dotted line.

In children with PA, serum DHEAS was mostly increased one to two-fold above the upper limit of normal (ULN) and did not exceed eight-fold ULN (Fig. 3A and Supplementary Table 3). PA girls had higher DHEAS than boys (*P* = 0.036) (Supplementary Fig. 1 and Supplementary Table 3). The single ACC case presented with serum DHEAS increased to 28-fold ULN. The majority of children with CAH had normal DHEAS levels (Fig. 3A and Supplementary Table 3).

A4 excess above two-fold ULN was typically observed in classic CAH patients (Fig. 3B and Supplementary Table 3). Mild-to-moderate A4 excess (one- to two-fold ULN) was observed in adolescent PCOS (42%), in some children with PA (24%) and in the ACC case. The boy with CD had severe A4 excess. In PA, A4 was higher in boys than girls (*P* = 0.0008) (Supplementary Fig. 1).

Both mild-to-moderate (one- to two-fold ULN) and severe (>two-fold ULN) excess of serum testosterone was found in a number of subjects with PA, PCOS and CAH (Fig. 3C and Supplementary Table 3). Testosterone excess was found in a minority of children with PA (10%), but two boys had severe testosterone excess; overall, serum testosterone did not differ between boys and girls with PA (Supplementary Fig. 1). In PCOS, testosterone excess was found in 63% of cases, but was severe in one only (Fig. 3C). In classic CAH, six subjects (50%) had testosterone excess, which was severe (five-fold elevation) in one adolescent girl.

## Discussion

Here, we have simultaneously measured the serum concentrations of the androgen precursors DHEAS and A4 as well as the active androgen testosterone in 487 consecutively recruited children and adolescents in a single tertiary pediatric referral center, covering a population of 5.5 million. The size of our cohort, comprising 199 children with biochemically confirmed androgen excess, enabled us to provide separate analyses of androgen excess pattern and severity in the pre- and post-pubertal age subgroups. Our study cohort included children presenting with clinical features of androgen excess, but also patients with suspicion of complex adrenal and gonadal pathologies, increasing the diverse and complex spectrum of underlying conditions. This enriched our study population for rare and very rare diagnoses. The study population was ethnically very diverse; in comparison to the local background population, there was some over-representation of South Asian and African-Caribbean ethnicities in PA children and particular over-representation of South-Asian ethnicity in the PCOS group. This may partially reflect the higher incidence of PCOS in South Asian subjects (16) as well as the proximity of our center to areas with higher ethnic diversity within the overall catchment area.

Previous studies have assessed serum androgens in pre-selected samples of children with distinct androgen excess conditions, mainly PA and adolescent PCOS. PA has been studied over the past 30 years in several case-control studies, in well described cohorts consisting of 10–100 study subjects of various ethnical–geographical backgrounds, but mainly focusing on metabolic consequences in PA ((1, 2, 17, 18) for comprehensive reviews), however, generally without providing full details on androgen metabolism including severity levels. Two studies reported immunoassay-based androgen concentrations in subjects with PA (19, 20). Virdis *et al*. reported elevated DHEAS levels in the majority of subjects up to 9-fold above the upper limit of age-matched controls, but normal A4 and testosterone excess only in most subjects studied (19). Voutilainen *et al*. did not report DHEAS levels, but found that unconjugated DHEA and A4 were up to 5-fold elevated in 18 PA subjects studied (20).

Adrenal androgen excess with elevated serum DHEAS above the age-specific reference range and a marginal increase in downstream androgens is recognized as a key feature of PA (2, 21); others suggested increased serum DHEAS appropriate for Tanner pubertal stage but increased when referring to age-specific reference ranges (22, 23). Of note, the pattern and severity of androgen excess in PA has not been analyzed in detail previously, neither how these differentiate PA from other conditions presenting with androgen excess such as CAH or other even rarer causes.

In our cohort, increased serum DHEAS was detected in the majority of children with PA while A4 and testosterone were increased only in a minority of subjects, despite clinical evidence of increased androgen action. DHEAS is an inactive metabolite with no affinity to the androgen receptor, suggesting that downstream conversion of de-sulfated DHEA to potent androgens could occur within the target tissues of androgen action. Our group and others have recently highlighted 11-oxygenated androgens as a major source of androgen excess in conditions like PCOS and CAH (24, 25, 26, 27). The adrenal P450 cytochrome 11β-hydroxylase (CYP11B1) enzyme efficiently converts A4 to 11-hydroxy-androstenedione, which can be further converted to 11-ketotestosterone (11KT), which has been shown to bind and activate the androgen receptor with similar potency to testosterone (24, 28, 29). In fact, 11KT has very recently been shown to feature prominently in children with PA (30), supporting the theory that androgenic effects in PA may be mediated via increased 11-oxygenated androgens. However, measurement of 11-oxygenated androgens does not yet form part of routine androgen assessment in childhood androgen excess and, thus, was not measured in our cohort.

Radioimmunoassays (RIAs) were employed in the past in studies assessing androgen metabolism in children, in particular the initial reports from the 1970s (31, 32), which are frequently referenced. RIAs are prone to cross-reactivity and less sensitive to lower circulating concentrations usually found in children. Our study has employed more sensitive and specific tandem mass spectrometry (LC–MS/MS) for the measurement of A4 and testosterone. Clinical biochemistry laboratories are now using mass spectrometry-based steroid panels with increasing frequency, which confronts the clinician often with more results than requested in the first place. Our findings can provide guidance for risk stratification in the assessment of childhood androgen excess.

Only recently, LC–MS/MS-based longitudinal measurements of androgens were reported in 40 obese girls with adolescent PCOS (33). Interestingly, that study reported elevated A4 and testosterone levels, but normal DHEAS compared to BMI-matched controls. This is in contrast to this study, but also our previous report on pre-menopausal PCOS women (12), where elevated serum DHEAS was the most common finding. We used standard reference values obtained from a lean cohort (14), which is probably most appropriate for daily clinical practice when referring to reference data.

In order to biochemically characterize common childhood androgen excess conditions, the patterns and severity of androgen levels may help the clinician to discriminate non-PA and non-PCOS pathology. Children with CAH presented predominantly with A4 and testosterone excess. Certainly, 17OHP elevation would be the key biochemical discriminator to detect of the most common form of CAH, 21-hydroxylase deficiency, and should be included in the diagnostic work-up of childhood androgen excess (1, 7). A basal serum 17OHP threshold of 2 ng/mL (6 nmol/L) has been shown to distinguish PA from non-classic CAH with high sensitivity and specificity (34). All the patients with classic CAH in our cohort were on hydrocortisone replacement therapy and hence severity and pattern of androgen excess will be distinct from androgen excess in newly diagnosed CAH patients. However, the serum androgen signatures observed in our CAH study sample form a very characteristic profile, which is helpful for the clinician in interpreting serum androgen profiles to differentiate CAH from other disorders associated with androgen excess.

Previous work has provided limited information on circulating androgens in girls with central precocious puberty (cPP) or IPM. Pubic or axillary hair development are rarely part of the spectrum in IPM (35), but have been reported in more than 60% of children with cPP (36). All girls with cPP (but none of the girls with IPM) in our cohort had additional (early) pubarche, which prompted the assessment of serum androgens. In our study, all five girls with IPM had mild DHEAS excess, which could reflect concomitant (or pre-existing) exaggerated adrenarche. Indeed it has been shown that higher adrenal androgens are linked to an earlier onset of puberty with a shorter peak height velocity (37). The underlying etiology of IPM is not well understood, and it is considered to be a benign, self-limiting condition (35). However, one might speculate that aromatization of excess androgens could expose the endometrium to higher levels of estrogens, enhancing the build-up of the endometrial lining, ultimately resulting in menarche.

ACC is an extremely rare entity in children, apart from certain geographical areas like Brazil where ACC is more prevalent due to a distinct founder mutation (p.R337H) of the *TP53* gene (38). The general rarity of ACC is also reflected in our study population with a single case diagnosed in 487 children over a 5-year study period. Interestingly, our patient also carried the Brazilian TP53 founder mutation, although the family was of South Asian origin. Based on a recent larger case series of children with ACC from the US (41 cases identified over a period of 60 years), androgen excess is the most frequently presenting steroid abnormality in childhood ACC (39). Details on serum androgen concentrations at presentation, however, are not widely reported in childhood ACC, presumably due to the rarity of the disease, complicated by historic nature of cases and varying assay methodologies. Based on our study and what is known from the literature, the findings certainly indicate that severe DHEAS excess beyond 8-fold ULN should prompt urgent further investigations to exclude ACC-related androgen excess.

In conclusion, we have undertaken a detailed analysis of pattern and severity of androgen excess in a large cohort of children of pre- and post-pubertal age. Our findings provide unique insights into the variety and frequency of childhood androgen excess conditions and the utility of combined measurements of serum DHEAS, A4 and testosterone. DHEAS excess is rare in CAH, which typically features A4 excess. In contrast, DHEAS excess is characteristic for PA and frequently found in adolescent PCOS; serum DHEAS increased above 8-fold ULN should prompt the clinician to perform further investigations to exclude sinister underlying pathology.

## Supplementary Material

Supplementary Fig. 1

Supplementary Table 1

Supplementary Table 2

Supplementary Table 3

## Declaration of interest

The authors declare that there is no conflict of interest that could be perceived as prejudicing the impartiality of this study.

## Funding

This work was funded by the Wellcome Trust (Investigator Award 209492/Z/17/Z to W A). J I is a National Institute of Health Research (NIHR) UK Clinical Lecturer and W A receives support from the NIHR Biomedical Research Centre Birmingham. The views expressed in this publication are those of the author(s) and not necessarily those of the National Health Service, the National Institute for Health Research, or the Department of Health.
