# Supplementary figures and images for: Causes, patterns and severity of androgen excess in 487 consecutively recruited pre- and post-pubertal children
Source: Eur J Endocrinol. 2018 Dec 19;180(3):213–21. doi: 10.1530/EJE-18-0854 (PMC6365673; doi:10.1530/EJE-18-0854)

**A** PA - girls (n=67)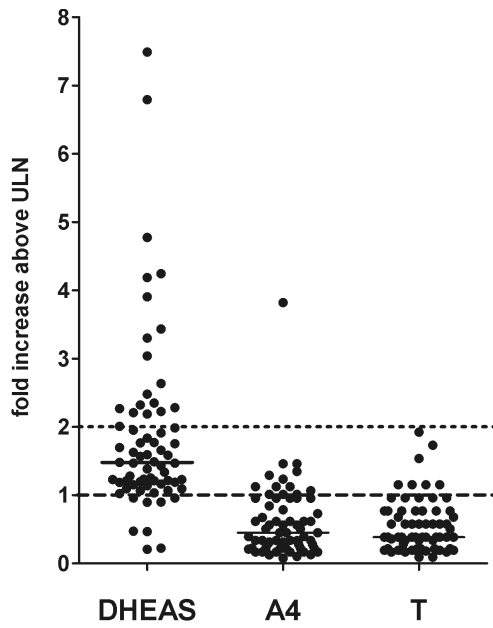**B** PA - boys (n=19)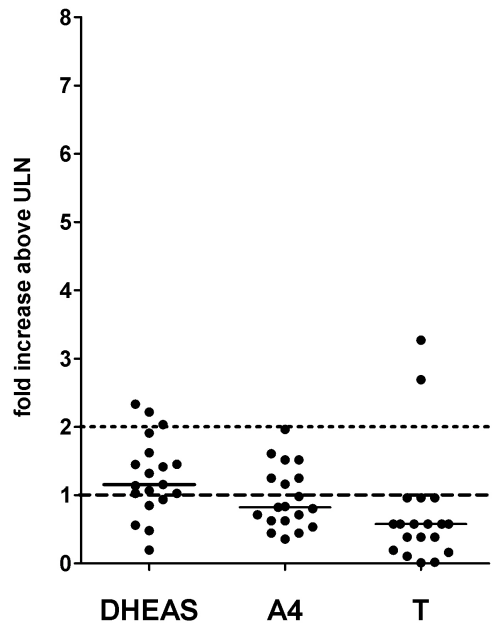**C** CAH - girls (n=8)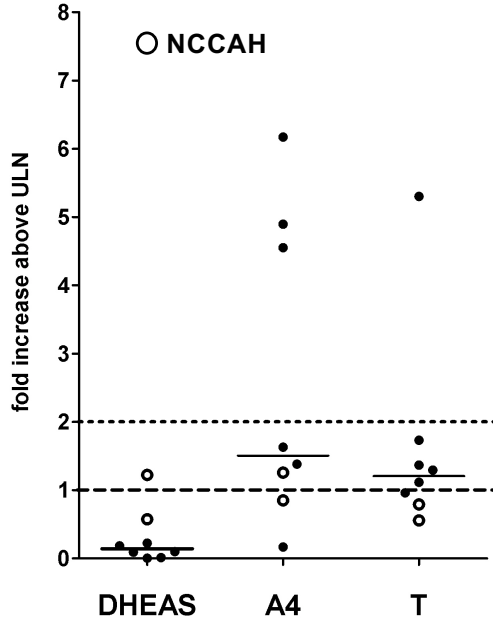**D** CAH - boys (n=6)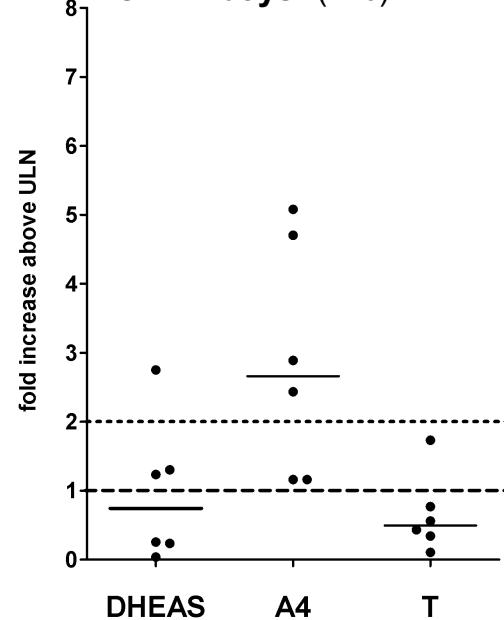**E** PCOS (n=24)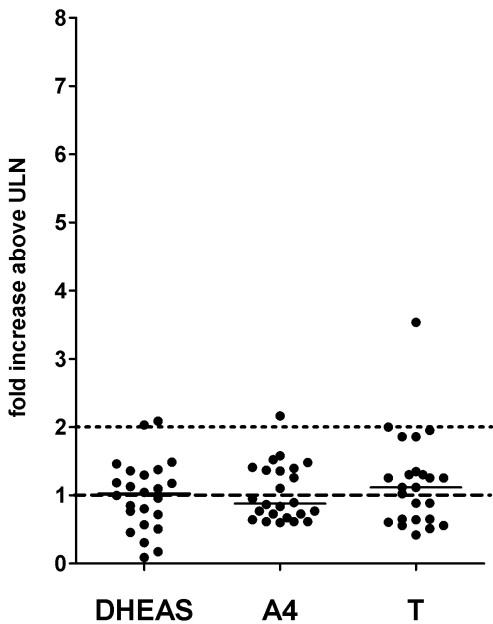**F** Disorders of Puberty (n=10)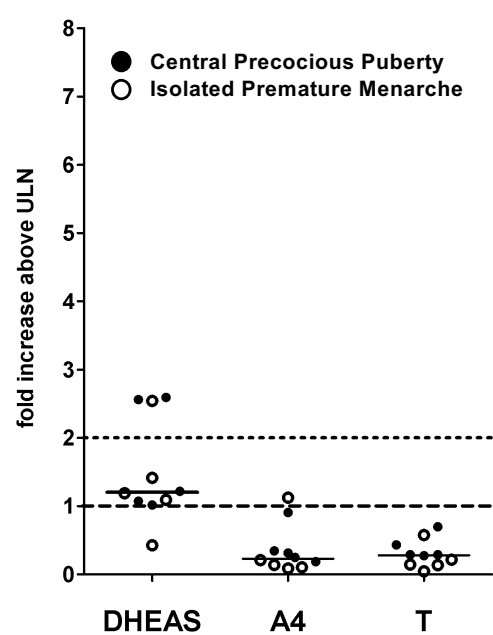

Supplement: Supplementary Fig. 1 [file supplementary_figure_1.pdf]
