# Supplementary material for: Causes, patterns and severity of androgen excess in 487 consecutively recruited pre- and post-pubertal children
Source: Eur J Endocrinol. 2018 Dec 19;180(3):213–21. doi: 10.1530/EJE-18-0854 (PMC6365673; doi:10.1530/EJE-18-0854)
Supplement: Supplementary Table 1 [file supplementary_table_1.pdf]

Suppl. Table 1: Miscellaneous diagnoses and features (as specified in Fig. 1) identified in 63 patients with at least one serum androgen concentration increased above the Tanner stage-specific reference range, but no conclusive diagnosis explaining androgen excess.

	**Pre-pubertal N=41**		**Post-pubertal N=22**	
	**Girls**	**Boys**	**Girls**	**Boys**
**Diagnoses/ features likely to be associated with or due to androgen excess** N=13	N=4	N=3	N=6	N=0
	Clitoromegaly, resolving (n=2)	Bilateral adrenal hemorrhages	Clitoromegaly (resolved)	
	Hypertrichosis, resolving	Germ cell tumor	Isolated acne (n=2)	
	Isolated thelarche	Anti-Müllerian-Hormone resistance	Oligomenorrhea (resolved)	
			Excessive sweating	
			Mood swings	
**Diagnoses/ features <u>not</u> likely to be associated with or due to androgen excess** N=50	N=13	N=21	N=13	N=3
	No diagnosis (n=5)	No diagnosis (n=9)	Simple Obesity (n=9)	Delay of growth and puberty
	Complex cloacal anomaly	Micropenis (n=2)	Road traffic accident	Buried penis, impalpable testes
	Electrolyte abnormalities	Klinefelter syndrome	Bardet Biedl Syndrome	Wiedemann Beckwith Syndrome
	Primary ovarian failure	Septo-optic dysplasia (n=2)	Atopic skin	
	Alström syndrome (n=2)	Pubertal arrest/ primary gonadal failure	Silver Russell Syndrome	
	CHARGE syndrome	CHARGE syndrome		
	Obesity	Hypospadias (n=2)		
	Aldosterone synthase deficiency	Alström syndrome		
		Prader Willi syndrome		
		Supra-sellar cyst		
