# Supplementary material for: Causes, patterns and severity of androgen excess in 487 consecutively recruited pre- and post-pubertal children
Source: Eur J Endocrinol. 2018 Dec 19;180(3):213–21. doi: 10.1530/EJE-18-0854 (PMC6365673; doi:10.1530/EJE-18-0854)
Supplement: Supplementary Table 2 [file supplementary_table_2.pdf]

Suppl. Table 2: Clinical signs and symptoms in 86 children presenting with premature adrenarche

	**Total** N=86 (100%)	**Girls** N=67 (79%)	**Boys** N=18 (21%)
Premature Pubarche	60 (71%)	50 (75%)	10 (56%)
Body odor	48 (56%)	38 (57%)	10 (56%)
Premature development of axillary hair	25 (29%)	21 (31%)	4 (22%)
Tall stature/ growth acceleration	21 (25%)	17 (31%)	4 (22%)
Acne	14 (17%)	13 (19%)	1 (6%)
Mood swings	14 (17%)	11 (16%)	3 (17%)
Breast development/ gynecomastia	12 (14%)	11 (16%)	1 (6%)
