# Supplementary material for: Causes, patterns and severity of androgen excess in 487 consecutively recruited pre- and post-pubertal children
Source: Eur J Endocrinol. 2018 Dec 19;180(3):213–21. doi: 10.1530/EJE-18-0854 (PMC6365673; doi:10.1530/EJE-18-0854)
Supplement: Supplementary Table 3 [file supplementary_table_3.pdf]

Suppl. Table 3: Severity of androgen excess in children 136 according to underlying diagnosis. Serum androgen data are presented as fold increased above the upper limit of normal (ULN) and as absolute serum concentrations, both expressed as median value and inter quartile ranges (IQR), if appropriate.

	**Premature Adrenache girls** n=67	**Premature Adrenarche boys** n=19	**Polycystic ovary syndrome** n=24	**Congenital adrenal hyperplasia girls** n=8	**Congenital adrenal hyperplasia boys** n=6	**Isolated premature menarche** n=5	**Central Precocious puberty** n=5	**Adreno-cortical carcinoma** n=1	**Cushing's disease** n=1
**Serum DHEAS**									
Fold increase above ULN (median, IQR)	1.5 (1.2; 2.2)	1.1 (1.0; 1.5)	1.0 (0.7, 1.3)	0.1 (0.1; 0.3)	0.7 (0.2; 1.3)	1.2 (1.0; 1.4)	1.2 (1.1; 2.7)	29.8	0.4
mol/L (median, IQR)	2.8 (2.2; 3.7)	3.6 (2.7; 5.3)	9.1 (6.0; 11.7)	0.7 (0.4; 2.8)	2.2 (0.9; 3.3)	2.6 (2.4; 3.3)	5.9 (2.5; 6.0)	15.8	2.3
**Serum androstenedione**									
Fold increase above ULN (median, IQR)	0.4 (0.3; 1.0)	0.8 (0.6, 1.3)	0.9 (0.7; 1.4)	1.5 (1.2; 4.6)	2.7 (1.5; 4.2)	0.1 (0.1; 0.2)	0.3 (0.3; 0.4)	1.5	3.8
nmol/L (median, IQR)	1.0 (0.6; 1.7)	1.2 (0.8; 1.7)	6.3 (5.1; 9.9)	7.8 (5.3; 25.7)	9.1 (2.8; 13.4)	1.0 (0.7; 1.1)	1.5 (1.2; 2.7)	4.5	6.4
**Serum testosterone**									
Fold increase above ULN (median, IQR)	0.4 (0.2; 0.8)	0.6 (0.3; 0.8)	1.1 (0.7, 1.3)	1.2 (0.9; 1.5)	0.5 (0.4; 0.7)	0.1 (0.1; 0.2)	0.3 (0.3; 0.4)	0.1	0.6
nmol/L	0.3	0.3	2.4	1.8	6.7	0.3	0.6	0.9	0.1

(median, IQR)	(0.2; 0.4)	(0.2; 0.5)	(1.4; 2.9)	(1.1; 2.0)	(1.5; 12.2)	(0.2; 0.3)	(0.4; 0.6)		
